# Risk profile for osteoradionecrosis of the mandible in the IMRT era

**DOI:** 10.1007/s00066-015-0875-6

**Published:** 2015-08-12

**Authors:** Gabriela Studer, Marius Bredell, Stephan Studer, Gerhard Huber, Christoph Glanzmann

**Affiliations:** 1 Department of Radiation Oncology, Head Neck Cancer Center, University Hospital Zurich, Raemistrasse 100, 8091 Zurich, Switzerland; 2 Department of Craniomaxillofacial and Oral Surgery, Head Neck Cancer Center, University Hospital Zurich, Zurich, Switzerland; 3 Department of Otorhinolaryngology, Head Neck Cancer Center, Head and Neck Surgery, University Hospital Zurich, Zurich, Switzerland

**Keywords:** Osteoradionecrosis, ORN, Risk factors, ORN, Preradiation surgery, Surgery-related ORN, Postoperative ORN, Osteoradionekrose, ORN, Risikofaktoren, ONR, Präradiotherapeutische Chirurgie, Chirurgiebedingte ORN, Postoperative ORN

## Abstract

**Background:**

The risk for osteoradionecrosis (ORN) of the mandible is positively related to bone volume exposed to > ~ 60 Gy. We hypothesized that in combined treatment, surgery may also be a risk factor.

**Patients and methods:**

The impact of mandibular surgery on ORN in locally disease-free IMRT cohorts was retrospectively analyzed.

**Results:**

Between October 2002 and October 2013, 531 of 715 patients with oral cavity cancer (OCC), mesopharyngeal cancer (MC), or salivary gland tumor were treated with the mandible bone exposed to ~ > 60 Gy (mean follow-up, 38 months; 7–143 months). Of the 531 patients, 36 developed ORN (7 %; 1.5 % with grade 3–4). The ORN rate in definitive IMRT MC (16/227) and in postoperative IMRT OCC patients with no mandibular surgery (3/46) was 7 % each; in OCC patients with mandibular surgery the rate was 29 % (15/60, *p* = 0.002). Marginal or periosteal bone resection was found to be a high risk factor (39 %, vs. 7 % followed by segmental or no resection, *p* < 0.0001).

**Conclusion:**

Marginal or periosteal bone resection of the mandible was identified as the highest ORN risk factor in our IMRT cohort.

## Abstract (de)

**Hintergrund:**

Das Risiko für die Entwicklung einer Kiefernekrose nach Radiotherapie (Osteoradionekrose, ORN) korreliert bekanntlich mit dem Knochenvolumen, das einer Dosis von ~ > 60 Gy ausgesetzt wurde. Hypothese war, dass die Chirurgie bei kombinierten Therapien ebenfalls einen Risikofaktor darstellt.

**Material und Methode:**

Der Einfluss chirurgischer Interventionen am Kiefer auf das Risiko einer ORN wurde in unserem lokal krankheitsfreien IMRT-Kollektiv retrospektiv analysiert.

**Ergebnisse:**

Zwischen Oktober 2002 und Oktober 2013 wurden 531/715 Patienten mit Mundhöhlenkarzinomen (OCC), Mesopharynxkarzinomen (MC) oder Speicheldrüsentumoren mit Dosen > 60 Gy am Kieferknochen behandelt (mittlere Beobachtungszeit 38 Monate; Spanne 7–143 Monate). Von 531 Patienten entwickelten 36 eine ORN (7 %; 1,5 % mit Grad 3–4). Die ORN-Rate nach definitiver IMRT bei MC (16/227) und nach postoperativer IMRT bei OCC ohne chirurgischen Eingriff am Kieferknochen (3/46) belief sich auf je 7 %, bei OCC-Patienten mit Operation am Kieferknochen auf 29 % (15/60; *p* = 0,002). Das höchste ORN-Risiko fand sich dabei für marginale oder periosteale Knochenresektionen (39 %, vs. 7 % nach segmentaler oder keiner Resektion; *p* < 0,0001).

**Schlussfolgerung:**

Marginale oder periosteale Kieferknochenresektionen gingen in unserer IMRT-Kohorte mit dem höchsten Risiko für die Entwicklung einer ORN einher.

## Introduction

The risk for osteoradionecrosis ORN) of the mandible is known to be positively related to radiation doses of > 60 Gy to the bone [1, 2]. Patients at the highest risk for ORN are expectedly those who undergo definitive radiation therapy with doses of ~ 70–72 Gy for oral cavity cancer (OCC) or large central or lateralized mesopharyngeal cancer (MC) close to the mandible.

Grade 3 ORN was shown to be substantially reduced (to < 1 %) by the use of intensity-modulated radiation therapy (IMRT) techniques [3–8]; grade 1–2 ORN (healing after limited surgery such as debridement, decortication, sequestrectomy, or spontaneously following conservative treatment) was reported to be < 10 % in patients at risk [9].

In our OCC cohort, a higher ORN rate was observed in the postoperative subgroup (60–66 Gy in 2.0 Gy/fraction) than in the definitive IMRT subgroups as well as in the postoperative IMRT MC cohort. This observation motivated us to analyze the impact of previous mandibular surgery on the risk for ORN.

Our hypothesis was that pre-IMRT surgery of the mandible may also be a risk factor for ORN.

## Patients and methods

We retrospectively analyzed a single-center OCC/MC/salivary gland tumor IMRT cohort with respect to ORN. Data were prospectively acquired in a StatView® database. Patients with ≥ 60 Gy to the mandible were included. The following potential risk cofactors were analyzed: surgical technique (no mandibular surgery vs. periosteal resection vs. segmental resection vs. marginal (horizontal or sagittal) resection), nicotine and alcohol abuse, carotid artery calcification depicted in the planning computed tomography, comorbidities [diabetes, chronic obstructive pulmonary disease (COPD), peripheral arterial occlusive disease, coronary heart disease, immunosuppression], age, gender, and T stage.

Approval from the local ethics committee was obtained for data evaluation of our IMRT cohort. The analysis start point was defined as the start of radiation.

### Radiation dose

Postoperative IMRT prescription doses ranged between 60 and 66 Gy in 2.0 Gy/fraction, definitive IMRT prescription doses ranged between 68 and 72 Gy, mostly 70 Gy or 69.6 Gy in 2.0 Gy or 2.11 Gy/fraction, five fractions per week. Attention was paid to avoid/minimize dose hot spots to the mandible. Early analyses of mandible dose–volumes of our first 73 IMRT patients at risk (included in the present analysis) were previously reported [3]. Dose–volume histograms (DVHs) of the mandibles exposed to > 60.0 Gy were calculated. The maximum dose (Dmax) is defined as the highest dose in 1 %.

Details of the IMRT schedules used have been provided in previous reports [10, 11].

### Systemic concomitant therapy

If indicated, cisplatin was concomitantly given (40 mg/m^2^/week). Since April 2006, cetuximab has been used for patients with contraindications for cisplatin chemotherapy (400 mg/m^2^ loading dose, followed by 250 mg/m^2^ 1 day/week). Age and/or comorbidity or early-stage disease were reasons not to add systemic therapy (15 %).

### Follow-up

All patients were regularly seen in our joint clinics 3–6 weeks after completion of IMRT, at the Departments of Otorhinolaryngology and Head and Neck Surgery or the Department of Craniomaxillofacial and Oral Surgery. Our Head Neck Cancer Center standards for patient assessment include physical examination with additional flexible fiber-optic endoscopy approximately every 2–3 months (MC) in the first follow-up (FU) year, every 3 months in the second to third year, and every 6 months in the fourth to fifth year.

### ORN grading

Out of several systems grading ORN, we used the system proposed by Glanzmann and Graetz [1]:

- Grade 1: Exposed bone without signs of infection for at least 3 months
- Grade 2: Exposed bone with signs of infection or sequester, but not grades 3–5
- Grade 3: ORN treated with mandibular resection, with satisfactory result
- Grade 4: ORN with persistent problems despite mandibular resection
- Grade 5: Death due to ORN

In addition, “grade 2–3” ORN was introduced to grade ORN treatable with limited surgery, i.e., debridement, decortication, sequestrectomy.

ORN of the mandible that occurred in relation to invasive interventions (dental implants, tooth extractions, reconstructive/rehabilitative osseous or soft tissue surgery) on gingiva and/or mandible regions previously irradiated with doses > 50 Gy was termed "postintervention ORN." Exposed hardware after surgery was not considered as ORN, except for cases with secondary exposure after previous coverage.

Pre-IMRT surgical interventions on the mandible that were regarded as inclusion criteria for the term "mandibular surgery" (MS) were:

- Periosteal stripping
- Marginal resection
- Segmental resection

The time delay between surgery and radiation ranged between 4 weeks to 7.5 weeks (mean 5.5).

### Dental care before/during/post radiation therapy

All patients underwent standardized dental care according to our in-house dental care protocol, which is characterized by its individual risk-adjusted approach [7].

### Statistical analysis

Statistical calculations were carried out using the statistics program implemented in StatView® (version 4.5; SAS Institute, Cary, NC). Univariate analyses were performed with a Cox proportional hazards regression model in StatView®. Actuarial survival data were calculated using Kaplan–Meier curves and log-rank tests implemented in StatView®; *p* values of < 0.05 were considered statistically significant.

Multivariate analysis was performed using the StatView® calculation program (Mantel–Cox log-rank test). DVHs curves were calculated using the Excel® (Office 2013) program.

## Results

### Cohort

We assessed 715 locally disease-free OCC/OC/SGT IMRT patients who were treated at our department between October 2002 and October 2013. The mean FU was 38 months (7–143; Table 1).

**Table 1:** Study cohort

Parameters	OCC	MC	SGT	Total
**Patients (** ***N*** **)**	202	441	72	715
**Gender** (% males)				75 %
**Mean age** (range)				62 (39–91)
**Mean FU** (range), months				38 (7–143)
**Histology**				
Squamous cell carcinoma	197	428	21	646
Adenocarcinoma	0	3	19	22
Adenoid cystic carcinoma	1	0	15	16
Others	4	10	17	31
**IMRT**				
Postoperative	145	97	69	311
Definitive	57	344	3	404
**T stage**				
T1	28	73	11	112
T2	59	150	15	224
T3	23	97	25	145
T4	65	112	19	196
Recurrence	27	9	2	38
**N stage**				
N0	72	75	29	176
N1-2b	86	232	38	270
N2c	31	107	4	142
N3	2	22	0	24
Recurrence	11	5	1	17
**5-Year disease control (postoperative/definitive)**				
Local	75 %/40 %	96 %/77 %	95 %/50 %	
Disease-free survival	62 %/36 %	80 %/61 %	73 %/0 %	
Overall survival	71 %/27 %	86 %/73 %	88 %/75 %	

*FU* follow-up, *OCC* oral cavity cancer, *MC* mesopharyngeal cancer, *SGT* salivary gland tumor.

Of 715 patients, 531 (74 %) were exposed to > 60 Gy to the mandible, representing the population at risk (Table 2).

**Table 2:** Cohort at risk (531/715 patients with > 60 Gy to the mandible bone; no ORN in bones exposed to < 60 Gy)

	Definitive IMRT	Postoperative IMRT	ORN events (*N* = 36)
**OCC,** > **60 Gy to the mandible**	1/52 (2 %)	18/106 (17 %)	**19/158 (12 %)**
ORN G1	0	0	**0**
ORN G2	0	0	**0**
ORN G2–3	0	15	**15**
ORN G3	0	0	**0**
ORN G4	1^a^	3^a^	**4**
**MC,** > **60 Gy to the mandible**	16/227 (7 %)	0/74 (0 %)	**16/301 (5 %)**
ORN G1	2	0	**2**
ORN G2	4	0	**4**
ORN G2–3	6	0	**6**
ORN G3	4	0	**4**
**SGT,** > **60 Gy to the mandible**			
ORN G2	0/3	1/69 (1 %)	**1/72 (1 %)**

^a^Four of the eight postintervention osteoradionecrosis (ORN) cases observed in our cohort developed grade 4 ORN.

*OCC* oral cavity cancer, *MC* mesopharyngeal cancer, *SGT* salivary gland tumor.

### DVHs of the mandible

**Fig. 1:**
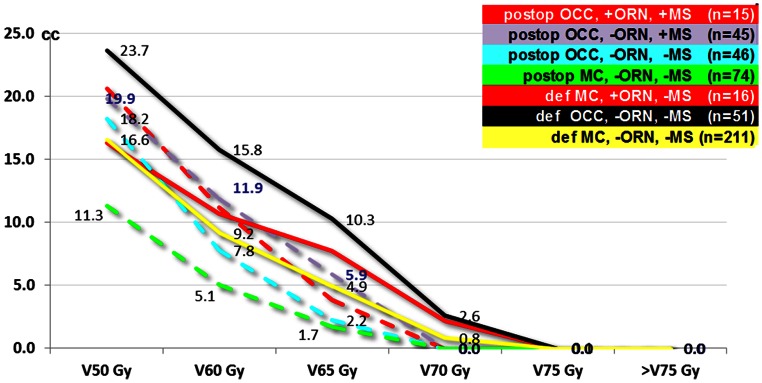
Mandible bone Dose–volume histograms (mean values, cc) of the following subgroups, all with the mandible exposed to > 60 Gy (*n* total = 458): -Postoperative IMRT in oral cavity cancer (*OCC*) patients with/without osteoradionecrosis (*±ORN*) who underwent mandibular surgery (*+MS*) or not (*−MS*) -Definitive IMRT in OCC patients (no MS, all in the cohort without ORN) -Definitive and postoperative IMRT in mesopharynx cancer (*MC*) patients ± ORN (none of them underwent mandibular surgery). *Red curves* represent the two cohorts with ORN (*+ORN*)*, dotted lines* postoperative IMRT groups*,* and *continuous lines* definitive IMRT groups

DVHs of the 531 patients at risk were calculated for all subgroups (Fig. 1). DVH mean values of postoperative IMRT MC patients were as expected significantly lower than those of postoperative IMRT OCC patients (*p* = 0.002). Postoperative IMRT OCC patients +/−ORN +/−mandible surgery (MS) and definitive IMRT MC patients without ORN had similar DVHs (ns). DVH values of definitive IMRT MC patients with ORN were nonsignificantly higher than those of patients with no ORN (unbalanced sample sizes: *n* = 16 vs. 211). Definitive IMRT OCC patients were characterized by the highest mandibular DVH values. Mean Dmax point doses ranged between 67.8 and 68.7 Gy in postoperative (ns) and between 72.4 and 73.6 Gy in definitive IMRT subgroups (ns).

### Osteoradionecrosis

The definitive IMRT subgroups (characterized by higher IMRT doses to the mandible, Fig. 1, continuous lines) showed a tendency to have lower ORN rates than the postoperative IMRT subgroups with lower IMRT doses (Fig. 1, dotted lines), with 17 ORN events in 282 patients (6 %) vs. 19 of 249 operated patients ( 7.6%, ns; Table 2), suggesting an additional risk factor impacting the risk for ORN other than radiation dose only.

We observed 5 % (36/715) ORN events of grade 1–4 in the entire cohort, and 7 % (36/531) in the cohort at risk (Table 2). All ORN events occurred in mandibles exposed to > 60 Gy.

Grade 1, 2, 2–3, 3, and 4 ORN events were observed in 2, 5, 21, 4, and 4 patients, respectively.

Of 36 ORN events, eight (22 %) developed as a consequence of invasive manipulation on previously with > 50 Gy irradiated bone areas (postintervention ORN): Four of eight events translated into complicated grade 4 ORN (pathological fracture, osteocutaneous fistula, osteomyelitis, persisting pain) following segmental resection or limited surgery for ORN. Two patients were treated with segmental resection (grade 3) with uncomplicated FU. Four of the eight postintervention ORN patients underwent postoperative IMRT (marginal resection in two patients, segmental resection in two patients).

All grade 3 ORN occurred in the definitive IMRT MC cohort: in two of four patients after 70 Gy in 2.0 Gy/fraction (D_max_ point doses 71.3 and 71.6 Gy, respectively), and in two of four following post-IMRT interventions.

The mean time from IMRT completion to ORN was 20 months (range, 1–104); the corresponding time for postintervention ORN was also 20 months (2–50).

### ORN in definitive IMRT OCC patients

**Fig. 2:**
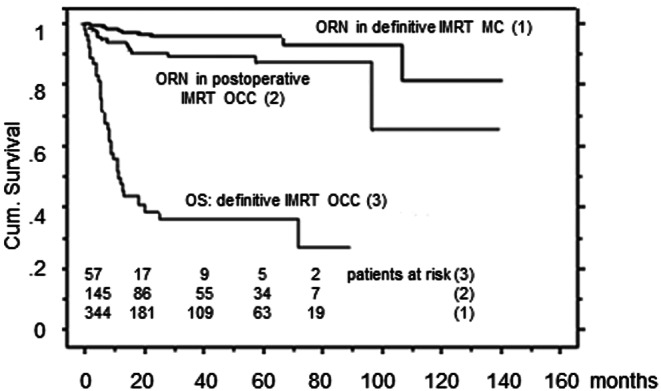
Overall survival (*OS*) of definitively irradiated OCC patients, and ORN-free survival in the postoperative IMRT OCC and definitive IMRT MC cohorts. *ORN* osteoradionecrosis, *OCC* oral cavity cancer, *MC* mesopharyngeal cancer

In this subgroup (Table 2) at highest risk (highest dose to largest mandible volume, Fig. 1), no ORN was diagnosed. Figure 2 shows Kaplan–Meier survival curves for ORN events following (1) definitive IMRT in MC patients and (2) postoperative IMRT in OCC patients: Most ORN events occurred early after IMRT completion, during the first 20 months. Curve (3) shows the overall survival (OS) of definitive IMRT OCC patients with no ORN (except for one postintervention ORN): only 14 of 57 patients reached 24 months.

### ORN in postoperative IMRT OCC patients

Tumor sites in operated OCC were the following: lip (*n* = 2), maxilla (*n* = 10), top/central part of the mobile tongue (*n* = 10), cheek (*n* = 12), lateral border of the mobile tongue (*n* = 29), and floor of the mouth/mandible (*n* = 82; see Table 2).

The postoperative subgroup who underwent mandibular surgery showed a statistically significantly increased risk for ORN as compared with operated OCC patients with no mandibular surgery [15/60 (14 G2–3 + 1 G4 (postintervention ORN, 25 %) vs. 3/46 (7 %), *p* = 0.0009]; the three (G2–3) ORN patients without mandibular surgery were all diagnosed with a carcinoma of the border of the mobile tongue and severe pre-existing comorbidity (nicotine ± alcohol abuse, COPD, polyneuropathy, postoperative wound healing problems, low body mass index, etc.). The D_max_ to the mandible was 66.7 Gy, 64.7 Gy, and 69.1 Gy, respectively.

Segmental resection was reasonably well tolerated (2 ORN G2–3 in 27 patients, 7 %, Table 3), while marginal or periosteal resection translated into a significantly higher ORN rate (14/37, 38 %, *p* < 0.0001; 13 G2–3).

**Table 3:** Types of mandibular surgery in postoperative IMRT patients at risk (> 60 Gy to the bone)

	ORN in patients with mandibular surgery and > 60 Gy			Total
	OCC	SGT	MC	
Type of mandibular surgery	(15/60, 25 %)	(1/3, 33 %)	(0/2, 0 %)	(16/65, 25 %)
Periosteal resection	5/13, 38 %	1/3, 33 %	0/1	**6/17, 35 %**
Marginal resection	8/20, 40 %	0	0	**8/20, 40 %**
Segmental resection	2/27, 7 %	0	0/1	**2/28, 7 %**

*MC* mesopharyngeal carcinoma, *OCC* oral cavity cancer, *SGT* salivary gland tumor.

Surgical reconstruction in 65 patients at risk who underwent mandibular surgery (Table 3) included insertion of a flap (48/65, 74 %), use of a free flap (40/48, 83 %), or titanium osteosynthesis plates (30/65, 46 %). In the 16 of 65 patients with ORN, a local or regional flap, a free vascularized flap, and bridging osteosynthesis plates only were used in 12 of 16 (75 %), 12 of 12 (100 %), and 6 of 16 (38 %) patients, respectively; the corresponding numbers in the remaining 49 of 65 patients who developed no ORN were similar, with 34 of 49 (71 %), 28 of 34 (82 %), and 24 of 49 (50 %), respectively (ns).

### ORN in MC patients

The rate of grade 1–3 ORN in MC patients was 4 % (16/441): 5 % (16/344) in the entire definitive IMRT MC cohort, 7 % (16/227) in patients with some aspect of the bone exposed to > 60 Gy (see Table 2). D_max_ point doses to the mandible of the 16 ORN-affected patients were a mean 73.6 Gy (range, 69.6–76.2 Gy). No ORN developed in 97 postoperative IMRT MC patients.

### ORN in SGT patients

Of 69 postoperative patients treated with > 60 Gy to the ascending ramus or angle of the mandible, one patient was diagnosed with grade 2 ORN: In this patient IMRT following periosteal resection and rarefication of the covering soft tissues resulted in a symptomatic necrosis of the condylar head (Table 2).

### Other potential risk cofactors

Age and gender were equally distributed in the ORN and non-ORN patients. T stage in ORN patients was distributed as follows: recurrent tumors (3), T1 (5), T2 (1), T3 (6), and T4 (13). The tested risk cofactor rates were tendentially nonsignificantly higher in the ORN than in the no-ORN cohort (nicotine 74 vs. 57 %, alcohol abuse 63 vs. 55 %, comorbidity 43 vs. 34 %, carotid artery calcification in planning computed tomography 31 vs. 26 %; unbalanced sample sizes: 36 with ORN, 680 without ORN). Risk factor grouping (none vs. nicotine ± alcohol, vs. comorbidity + nicotine or alcohol) also resulted in nonsignificant differences in uni- and multivariate analyses.

## Discussion

Previous surgery to the mandible, and mainly the type of surgery, was found to be the most important ORN risk parameter following IMRT.

In addition, this analysis confirmed that doses of < ~ 60–65 Gy to the mandible represent the lower threshold for any risk of ORN. An ORN rate (grade 2–3) of 39 % was found for postoperative IMRT patients at risk with floor of the mouth carcinoma or mandible infiltrating carcinoma who underwent previous periosteal or marginal mandibular resection (Table 3). This was statistically significantly different compared with postoperative IMRT OCC patients with no or segmental resection (7 %, each). A plausible explanation for this finding is that the mandible is more dependent on the periosteum for its blood supply than on the inferior alveolar neurovascular bundle, especially in older individuals [12]. During surgery, significant periosteal stripping or resection may take place with or without bone removal. This will lead to a denuded mandibular cortical plate with compromised healing capacity. In segmental resection cases the surgery is more extensive and mostly requires a composite free vascularized graft with a well-nourished bone flap with an unharmed periosteum and adequate soft tissue coverage.

Table 4 summarizes the ORN rates of our MC/OCC cohorts based on combined surgical and IMRT-related risk parameters. The fact that no ORN developed in 74 patients with postoperative MC vs. 17 % in 106 patients with postoperative OCC is explained by (a) the lower dose to the bone (DVH) and (b) only 2 of 74 cases underwent mandibular surgery in the MC subgroup.

**Table 4:** ORN rates related to mandibular surgery (MS) and DVH in OCC/MC patients at risk (> 60 Gy to the bone, *n* = 459; see also corresponding DVHs, Fig. 1.

DVH: V50–60–65–70–75 Gy	MC postop no MS (72)	MC postop with MS (2) (1 segmental, 1 periosteal)	OCC postop with MS (60)		OCC postop no MS (46)	MC def. no MS (227)	OCC def. no MS (52)
			Periosteal or marginal (33)	Segmental (27)			
~ 11–5–2–0–0 cc	0 %	0 %					
~ 17–10–4–1–0 cc			39 %	7 %	7 %	7 %	
~ 23–15–10–2.5–0 cc							(0 %)^*^

*DVH* dose–volume histogram, *MC* mesopharyngeal carcinoma, *OCC* oral CAVITY Cancer, *postop* postoperative IMRT (prescription dose 60–66 Gy), *def.* definitive IMRT (prescription dose 70–72 Gy).

* see also Figure 2: short OAS of this subgroup

The observation of no ORN event (but one postintervention ORN) in the cohort at highest risk, i.e., definitively irradiated OCC, seems to be sufficiently explained by the fact of the survival time being too short to experience ORN (only ~ 35 % surviving 20 months, Fig. 2). Poor outcome following definitive irradiation of OCC also in the IMRT era was previously reported; however, data on this topic are scant [13], which may partly be explained by a negative selection of OCC patients referred for definitive radiation (elderly, comorbid patients with large/inoperable tumors). For the small subgroup (*n* = 14) who survived 2 years, we cannot exclude an ORN rate of approximately 22 % (according to the statistical "rule of three" to estimate the probability of adverse events in small sample sizes with few events, giving the upper limit of the 95 % confidence interval of the probability: 3/14 = 22 %).

The positive relationship between radiation dose–volumes and the risk for ORN is known, and the reduced ORN risk by using mandible-sparing IMRT techniques was confirmed in several reports [3, 5–8]. Only scant information is available on the ORN risk in the postoperative setting, which is characterized by lower radiation doses to the mandible than in the definitive radiation setting. Table 5 gives an overview of the ORN events in our patients related to the treatment sequence.

Korean researchers reported on OCC/MC patients irradiated postoperatively with conventional three-dimensional radiation techniques. The authors found a significantly higher ORN rate in 59 patients with previous mandibular surgery than in 139 patients with no mandibular surgery (13.6 vs. 3.6 %, *p* = 0.01), with a latency from radiation to onset of ORN of a mean 22 months—comparable to our own results—and an ORN grade 3 rate of 38 % (5/13) [14].

Comorbidity was not found to be statistically significantly influencing the ORN risk in our cohort, which may be due to sample size imbalance. Gevorgyan et al. found no association between the severity of 14 ORN cases (grade 1–3, similar to our classification, 8/14 following definitive radiation) and gender, age smoking, alcohol abuse, TN stage, RT technique, or performance status [8]; they reported a significantly lower ORN incidence following IMRT compared with conventional techniques (*p* < 0.015).

With respect to ORN events in postoperatively irradiated parotid gland tumor patients, temporal bone ORN after surgery was reported by Leonetti et al. [15], who assessed 221 patients divided into the following groups: (1) parotidectomy only; (2) parotidectomy with mastoidectomy; and (3) parotidectomy with subtotal petrosectomy. The overall incidence of temporal bone ORN in group 1 was 2/106 (2 %); in group 2, 8/64 (13 %); and in group 3, 0/51 (0 %; radiation techniques not indicated). Because the horizontal and angular aspect of the mandible bone is at highest risk, ORN is a rare event following postoperative parotid gland tumor irradiation. This low susceptibility to ORN may be partially explained by the fewer surgical interventions performed in this area, the absence of teeth, and adequate soft tissue coverage by the masticatory muscles.

In summary, the following conclusions can be drawn from the present results:

1. ORN or postintervention ORN developed only after IMRT doses of > 60 Gy to the mandible (36/531, 7 %).
2. The grade 3 ORN rate following IMRT in patients at risk (2/531 with > 60 Gy to the mandible) was < 0.1 % (postintervention ORN excluded).
3. The subgroup at highest risk for ORN was floor of the mouth/mandible carcinoma patients treated with previous periosteal or marginal mandibular resection (39 % grade 2–3 ORN).
4. Postoperative IMRT OCC patients with a dose of > 60 Gy to the mandible who underwent previous segmental or no mandibular resection had significantly lower ORN rates (7 % each, *p* < 0.0001).
5. The ORN rate of definitive IMRT MC patients with > 60 Gy to the bone was 7 %.
6. Definitive IMRT OCC patients as the subgroup at theoretically highest risk for ORN were characterized by a very poor outcome with survival time in most patients shorter than the onset of ORN (negative selection of patients not suitable for surgery); this likely explains the low ORN rate in this subgroup.
7. Postoperative IMRT MC patients represented the subgroup at lowest risk for ORN (0 %), explained by their lowest mandibular DVH values and only 2/97 cases with previous mandibular surgery.
8. Half the patients with postintervention ORN developed grade 4 ORN (4/8)

**Table 5:** Overview of the ORN events (crude rates) in the cohort at risk, related to surgery and IMRT sequence

	Patients with mandible exposed to > 60 Gy (*N* = 531)							
IMRT sequence	Definitive IMRT (*n* = 282)			Postoperative IMRT (*n* = 249))				
Site	SG	OCC	MC	MC	SG		OCC	
No mandibular surgery (*n*)	3/3	52/52	227/227	72/74		66/69		46/106
With mandibular surgery (*n*)	0	0	0	2/74	3/69		60/106	
**ORN**	**0 % (0/3)**	**2 % (1/52)**	**7 % (16/227)**	**0 % (0/74)**	**33 % (1/3)**	**0 % (0/66)**	**25 % (15/60)**	**7 % (3/46)**

*MC* mesopharyngeal carcinoma, *OCC* oral cavity cancer, *SG* salivary gland tumor.

## Conclusion

Periosteal or marginal mandibular resection was the statistically significantly highest ORN risk factor, translating into a 39 % ORN rate grade of 2–3, vs. 7 % following definitive IMRT or postoperative IMRT with no or segmental resection.

Consequently, the radiation dose to the mandible should be minimized in patients at high risk for ORN.
